# Human SHBG mRNA Translation Is Modulated by Alternative 5′-Non-Coding Exons 1A and 1B

**DOI:** 10.1371/journal.pone.0013844

**Published:** 2010-11-04

**Authors:** Tomàs Pinós, Anna Barbosa-Desongles, Antoni Hurtado, Albert Santamaria-Martínez, Inés de Torres, Jaume Reventós, Francina Munell

**Affiliations:** 1 Institut de Recerca Hospital Universitari Vall d'Hebrón, Barcelona, Spain; 2 Centro de Investigación Biomédica en Red de Enfermedades Raras (CIBERER), Valencia, Spain; 3 Servei d'Anatomía Patològica, Hospital Universitari Vall d'Hebrón, Barcelona, Spain; University of Barcelona, Spain

## Abstract

**Background:**

The human sex hormone-binding globulin (SHBG) gene comprises at least 6 different transcription units (TU-1, -1A, -1B, -1C, -1D and -1E), and is regulated by no less than 6 different promoters. The best characterized are TU-1 and TU-1A: TU-1 is responsible for producing plasma SHBG, while TU-1A is transcribed and translated in the testis. Transcription of the recently described TU-1B, -1C, and -1D has been demonstrated in human prostate tissue and prostate cancer cell lines, as well as in other human cell lines such as HeLa, HepG2, HeK 293, CW 9019 and imr 32. However, there are no reported data demonstrating their translation. In the present study, we aimed to determine whether TU-1A and TU-1B are indeed translated in the human prostate and whether 5′ UTR exons 1A and 1B differently regulate SHBG translation.

**Results:**

Cis-regulatory elements that could potentially regulate translation were identified within the 5′UTRs of SHBG TU-1A and TU–1B. Although full-length SHBG TU-1A and TU-1B mRNAs were present in prostate cancer cell lines, the endogenous SHBG protein was not detected by western blot in any of them. LNCaP prostate cancer cells transfected with several SHBG constructs containing exons 2 to 8 but lacking the 5′UTR sequence did show SHBG translation, whereas inclusion of the 5′UTR sequences of either exon 1A or 1B caused a dramatic decrease in SHBG protein levels. The molecular weight of SHBG did not vary between cells transfected with constructs with or without the 5′UTR sequence, thus confirming that the first in-frame ATG of exon 2 is the translation start site of TU-1A and TU-1B.

**Conclusions:**

The use of alternative SHBG first exons 1A and 1B differentially inhibits translation from the ATG situated in exon 2, which codes for methionine 30 of transcripts that begin with the exon 1 sequence.

## Introduction

Sex hormone-binding globulin (SHBG) is a dimeric glycoprotein that transports sex steroids in the blood and regulates their access to target tissues [1]. The human SHBG gene is located in the short arm of chromosome 17 (17p13.1), contains at least 6 different transcription units, which are constituted of a common region that spans exons 2 to 8, and 6 alternative first exons [2]. These exons are named 1, 1A, 1B, 1C, 1D and 1E, following their 5′ to 3′ orientation on the positive strand of chromosome 17, and are all spliced to exon 2 using the same 3′ splice site [2]. Exons 1 and 1A (previously known as alternative exon 1) were the first to be characterized and have been extensively studied [1], [3], [4], [5], [6], [7], [8]. Exon 1 encodes a signal peptide and is responsible for production of plasma SHBG by the hepatocytes. TU-1 is regulated by promoter 1 sequence that contains several binding sites for liver-enriched transcription factors [8], [9]. TU-1A begins with the exon 1A sequence, which does not contain an ATG in frame with the SHBG coding sequence. It has been proposed that TU-1A initiates translation at the first ATG in frame of exon 2, which codes for methionine 30 of transcripts beginning with exon 1 [7], [8]. It has also been described that TU-1A is regulated by an alternative promoter sequence [6], [8] that proved to be very active when transfected in the GC2 mouse germ cell line [7]. The presence of full-length TU-1A has been demonstrated in human testis, liver, prostate, breast, and brain tissue, in human cancer cell lines derived from prostate (LNCaP) and breast (MCF-7), and in the testis of mice containing the 11-kb human SHBG transgene [5], [8].

Exons 1B, 1C, 1D (also known as exon 1N) and 1E, have been recently identified and described in human prostate tissue, in LNCaP, PC3, and PZ-HPV7 prostate cancer cell lines, and in several cancer cell lines originating in other tissues [2], [5]. As occurred with exon 1A, exons 1B, 1C, 1D and 1E do not contain an ATG in frame with the SHBG coding sequence. Therefore, as is the case of exon 1A, exons 1B, 1C, 1D, and 1E are 5′ untranslated regions (5′UTRs) of their corresponding TUs and might also initiate translation at the first in-frame ATG of exon 2 [2]. Full-length TU-1B transcripts have been detected in LNCaP cells and in human prostate tissue [2], TU-1D in LNCaP and MCF-7 cell lines [5], and TU-1C in the rhabdomyosarcoma CW 9019 and neuroblastoma *imr* 32 cell lines [2].

Alternative promoter usage has been shown to enable diversified transcriptional regulation in different cellular conditions or development stages [10], [11], and along with alternative splicing are the primary sources of 5′UTR transcript diversity [12]. Estimates of the number of genes with alternative 5′UTRs vary from 12% to 22%, while those of alternative promoter usage range from 10% to 18% [12]. Recent studies have shown that 5′UTRs play an important role in regulation of gene expression in a variety of organisms (microbes, plants, and animals) [13]. 5′UTR-mediated regulation has been shown to modulate gene expression through stimulatory and inhibitory mechanisms [13], [14], influencing the mRNA secondary structure, mRNA stability and translation efficiency [4], [13], [14], [15]. Specifically, it has been shown that occurrence of start codons and open reading frames upstream of the authentic start codon (uAUGs and uORFs, respectively) may affect mRNA translation [12], [14], [16], [17]. The different SHBG 5′UTRs exhibit many of the features associated with cellular mRNAs, whose expression is tightly controlled at the level of translation, including uORFs and thermodynamically stable predicted RNA structures [2], [5]. The present study determines the impact of exon 1A and 1B 5′UTRs on SHBG translation.

## Results

### Identification of potential translation regulatory elements in SHBG exons 1A and 1B

To investigate whether SHBG TU-1A and TU-1B are translated and how their alternative first exons modulate SHBG translation, we analyzed these sequences to identify potential translation regulatory elements, such as uAUGs, G/C-rich sequences, and stable secondary structures within 5′UTRs, known to serve as effective barriers to the scanning ribosomes [18], [19], [20].

**Figure 1:**
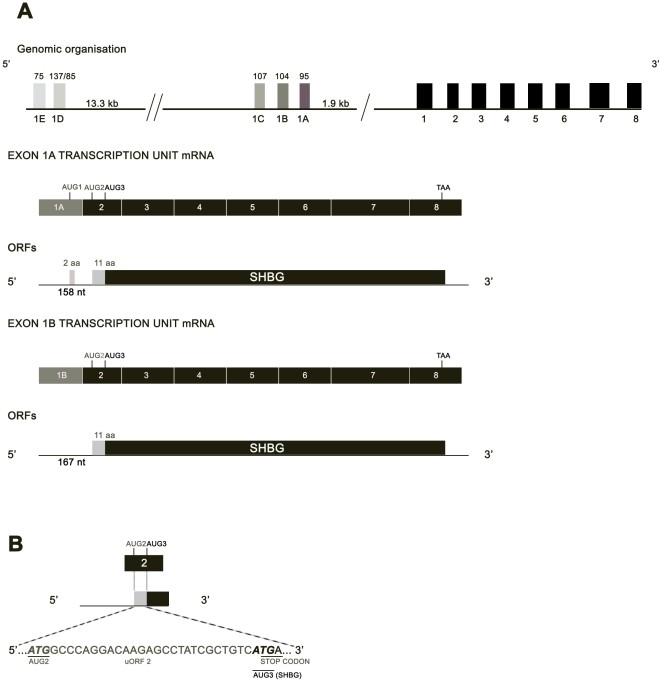
Identification of uORFs in TU-1A and TU-1B. A) The upper panel contains a schematic representation of the SHBG gene. Coding exons are shown in black boxes and noncoding alternative first exons are in gray boxes, with their respective sizes (in bp) indicated above. Below are depicted the exon organization of mature mRNAs of TU-1A and 1B and the corresponding ORF organization. uAUGs (AUG1 and 2) are written in gray while SHBG AUG is written in black (AUG3). uORFs are indicated in gray boxes while SHBG ORF is shown in a black box. AUG1 generates an uORF of 2 codons while AUG2 generates an uORF of 11 codons. B). Exon 2 scheme, showing AUG2 and 3. AUG2 starts a uORF whose stop codon overlaps the SHBG translation start codon (AUG3).

**Figure 2:**
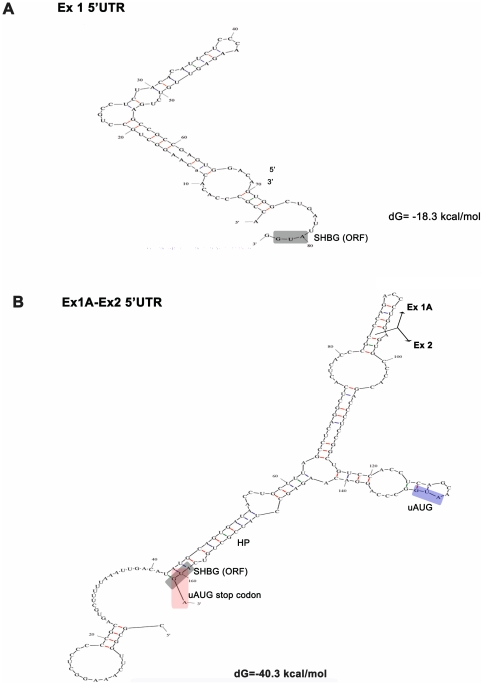
Predicted secondary structure for 5′UTR sequences of TU-1 and TU-1A. M-fold prediction of the 5′UTR mRNA secondary structure of TU-1 (A) and TU-1A (B). Predicted thermodynamic stability is shown, and the first SHBG codon is marked with a grey box. Junction between exons 1A and 2 is indicated with a solid black line in B. The uAUG is shown in a blue box in B. The predicted major hairpin is also indicated (HP).

**Figure 3:**
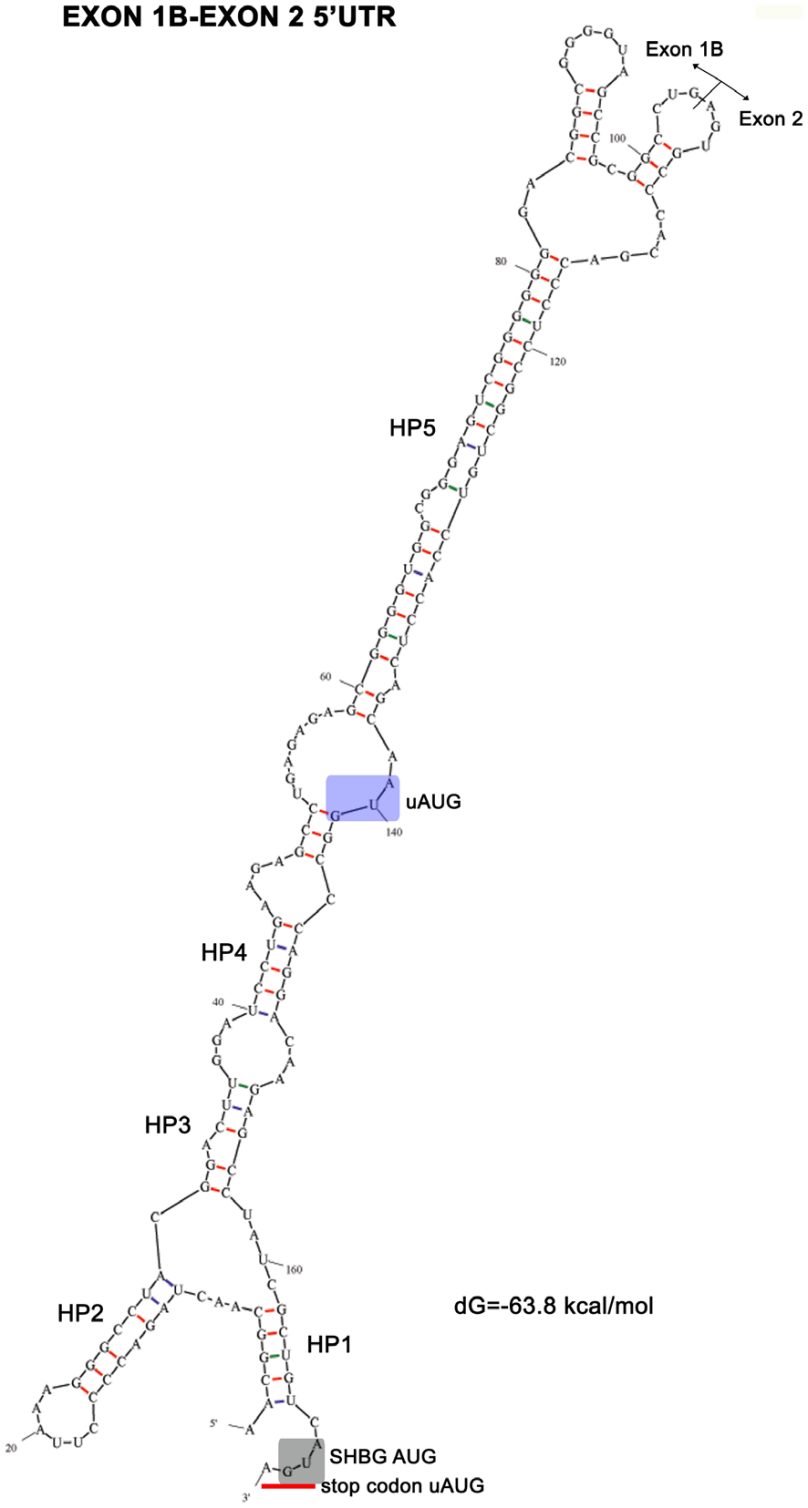
Predicted secondary structure for 5′UTR sequences of TU-1B. M-fold prediction of the 5′UTR mRNA secondary structure of TU-1B. Predicted thermodynamic stability, the first SHBG codon, junction between exons 1B and 2, and the uAUG are indicated as in Figure 2. The presence of hairpins is also indicated (HP1, HP2, HP3, HP4 and HP5).

SHBG TU-1 contains a short 5′UTR of 79 nucleotides without an uAUG (Genbank EU352659) [1], [5]. In contrast, TU-1A has a 5′UTR of 158 nucleotides with 2 uAUGs (one in the exon 1A sequence and the other in the exon 2 sequence) (Genbank X16351.1; Figure 1A), and TU-1B contains a 5′UTR of 167 nucleotides with 1 uAUG (the one in the exon 2 sequence) (Figure 1A). The uAUG of exon 1A produces an uORF of only 2 codons, whereas the uAUG found in exon 2 of both TU-1A and TU-1B generates an uORF of 11 codons, and the corresponding stop codon overlaps with the predicted translation start site of the SHBG ORF (Figure 1B). Additionally, both the M-fold and P-fold programs predict a higher level of thermodynamic stability for the secondary structures of both TU-1A [dG = −40.3 kcal/mol (M-fold); [2] and dG = −44.02 kcal/mol (P-fold), with 58.22% G/C] and TU-1B [dG = −63.8 kcal/mol (M-fold); [2] and dG = −69.9 kcal/mol (P-fold), with 67.9% G/C], in comparison with TU-1 [dG = −18.3 kcal/mol (M-fold) and dG = −18 kcal/mol (P-fold), with 58.2% G/C] (Figure 2A, B and Figure 3; Table 1). According to the M-fold program, in the TU-1A 5′UTR, the exon 2 translation start site is found at the end of a stable hairpin (HP, dG = −8.50 kcal/mol; Figure 2B), while in the case of the TU-1B 5′UTR, it is found within a loop preceded by 4 minor hairpins (HP 1, 2, 3, and 4; Figure 3), and one major hairpin (HP 5, dG = −26.20 kcal/mol; Figure 3).

**Table 1:** SHBG classification of alternative 5′UTRs.

5′ UTRExons	5′ UTRLength	5′TOP	ΔG (kcal/mol)≤−34.5/>−34.5	uAUGs	CARTclass
Exon 1	79 nt	No	−18.3/−18.0	0	III
Exon 1A-2	158 nt	No	−40.3/−44	2	I
Exon 1B-2	167nt	No	−63.8/−69.9	1	I

### Absence of SHBG protein translation in human prostate

**Figure 4:**
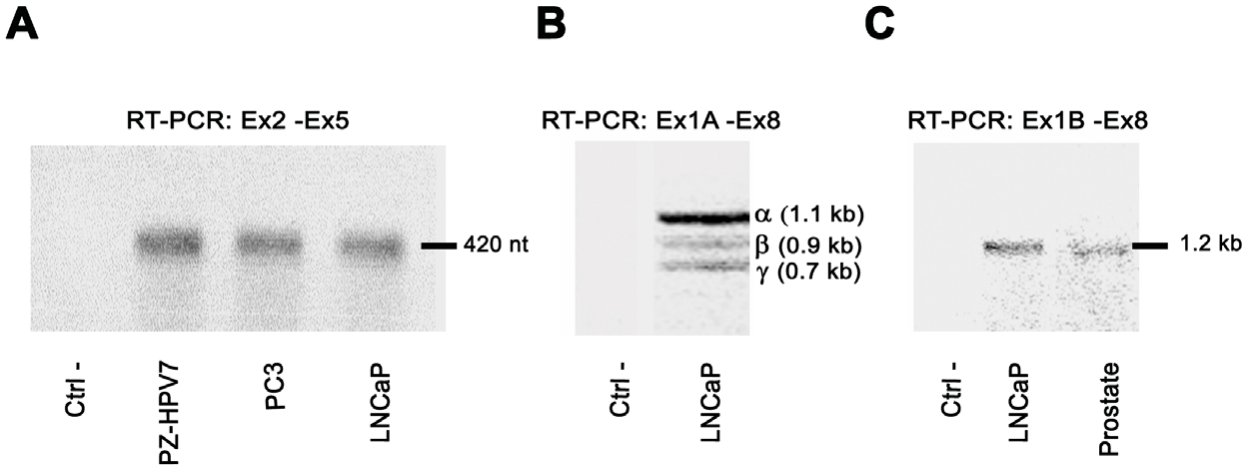
SHBG expression in human prostate cell lines. A) RT-PCR analysis of SHBG expression in human prostate-derived cell lines, using primers that amplify a region common to all SHBG mRNA isoforms (Ex2-Ex5). B) RT-PCR analysis (Ex1A-Ex8) of exon 1A transcripts in LNCaP cells. The different bands observed correspond to: α (full length transcript), β (skipping of exon 7) and γ (skipping of exons 6 and 7). C) As previously reported [2], full-length TU-1B transcripts are detected by RT-PCR analysis (Ex1B-Ex8) in human prostate tissue and LNCaP cells. Negative controls (Ctrl) are performed with water instead of cDNA.

**Figure 5:**
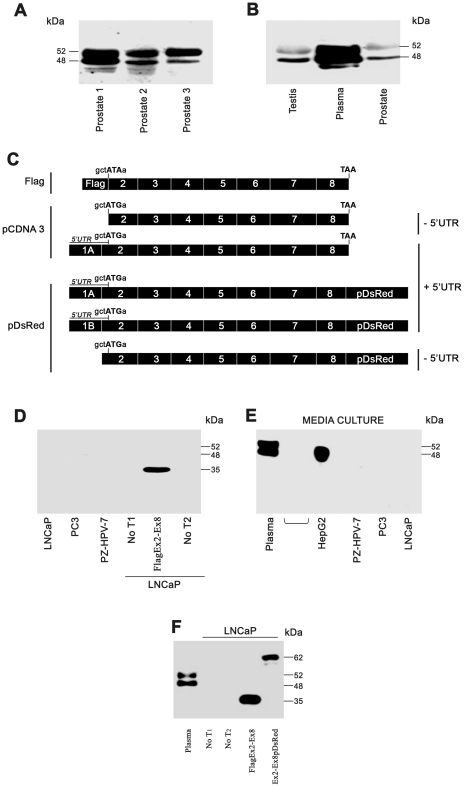
Western blot analysis of SHBG protein in human prostate. A–B) Two bands sized 52- and 48-kDa were detected in human prostate tissue using the SHBG 11F11 antibody (A) and were identical in size to those detected in human plasma and human testis (B). C) SHBG constructs used to transfect LNCaP cells. In the Flag-Ex2-Ex8 construct, SHBG ATG was mutated to ATA to avoid disturbing the Flag translation start site. The nucleotides involved in the SHBG translation start site are shown in all the constructs. D−E) Western blot analysis of the SHBG protein using the 11F11 antibody in transfected and non-transfected human prostate-derived cell lines (D), and in the supernatant (E). In (E), plasma and the hepatocarcinoma cell line (HepG2) were used as positive controls for secreted SHBG. F) Western blot analysis of LNCaP cells transfected or not with Flag-SHBG and pDsRed-SHBG constructs.

In the present study, mRNAs corresponding to a region (Ex2-Ex5) common to all SHBG TUs (Figure 4A), as well as exon 1A and exon 1B full-length transcripts (Figure 4B, C) were detected by RT-PCR. We then performed western blot using the SHBG 11F11 antibody (which recognizes the aminoacids encoded by exon 7 and the beginning of exon 8; Figures S1, S2) in order to detect the SHBG protein translated from these human prostate mRNAs. Two bands were detected in human prostate tissue, which were identical in size (52 and 48 kDa) to those seen in plasma and testis (Figure 5A, B). However, when total protein extracts from LNCaP, PC3, and PZ-HPV7 were analyzed, no SHBG protein was detected, except in LNCaP cells transfected with the Flag-_EX2-EX8-_SHBG construct, used as a positive control of SHBG translation, (Figure 5D), in which a protein band approximately 35 kDa in size was observed. This size matched well with the predicted molecular weight of the 345 amino acids of the SHBG protein fused to the 8 amino acids of the Flag tag (Figure 5C). These results support the idea that the SHBG protein is not present or at least cannot be detected by this technique in LNCaP, PC3, and PZ-HPV-7 cells. We then checked the capacity of these prostate cell lines to secrete SHBG, using the HepG2 hepatocarcinoma cell line as a positive control. Western blot analysis of supernatants from the 4 cell lines grown without FBS for 72 h demonstrated the protein exclusively in HepG2 culture medium, and showed a molecular size (52 and 48 kDa) identical to that of the protein found in plasma (Figure 5E). The absence of SHBG protein in prostate cell lines suggested that the protein found in prostate human tissue corresponded to plasma SHBG. Deglycosylation of SHBG extracted from prostate tissues reduced both bands to a lower molecular size of approximately 37 kDa (data not shown), confirming that both bands corresponded to the SHBG monomers characteristic of the secreted protein and were likely of blood origin.

### 5′ UTR sequences modulate SHBG gene expression

To test whether absence of the SHBG protein in prostate cancer cell lines resulted from reduced translation due to a modulatory action of the 5′UTR sequences of exon 1A and 1B on the translation efficiency from the first ATG in frame of exon 2, LNCaP cells were transfected with several SHBG constructs with and without exon 1A and exon 1B 5′UTR sequences (Figure 5C).

When LNCaP cells were transfected with the SHBG_EX2-EX8_-pDsRed construct, which did not include any SHBG 5′UTR sequence, an immunoreactive band of approximately 62 kDa in size was detected. This band matched well with the predicted molecular weight of the SHBG-pDsRed fusion protein (345 aa from the SHBG protein plus 225 aa from the C-terminus pDsRed-fused tag) (Figure 5C). Again, no band was detected in LNCaP non-transfected cells, while the 35 kDa band was observed when these cells were transfected with the Flag-_EX2-EX8-_SHBG construct (Figure 5F). Additionally, when LNCaP cells were transfected with the tag-less Ex2-Ex8-pCDNA 3.1 construct (without any SHBG 5′UTR sequence), a band of approximately 35 kDa was detected. Therefore, the SHBG protein is only detected in LNCaP transfected cells, and the differing molecular weight of the immunoreactive bands was caused by the presence and length of the tags fused to the SHBG protein, which is an additional indicator of the specificity of the antibody.

**Figure 6:**
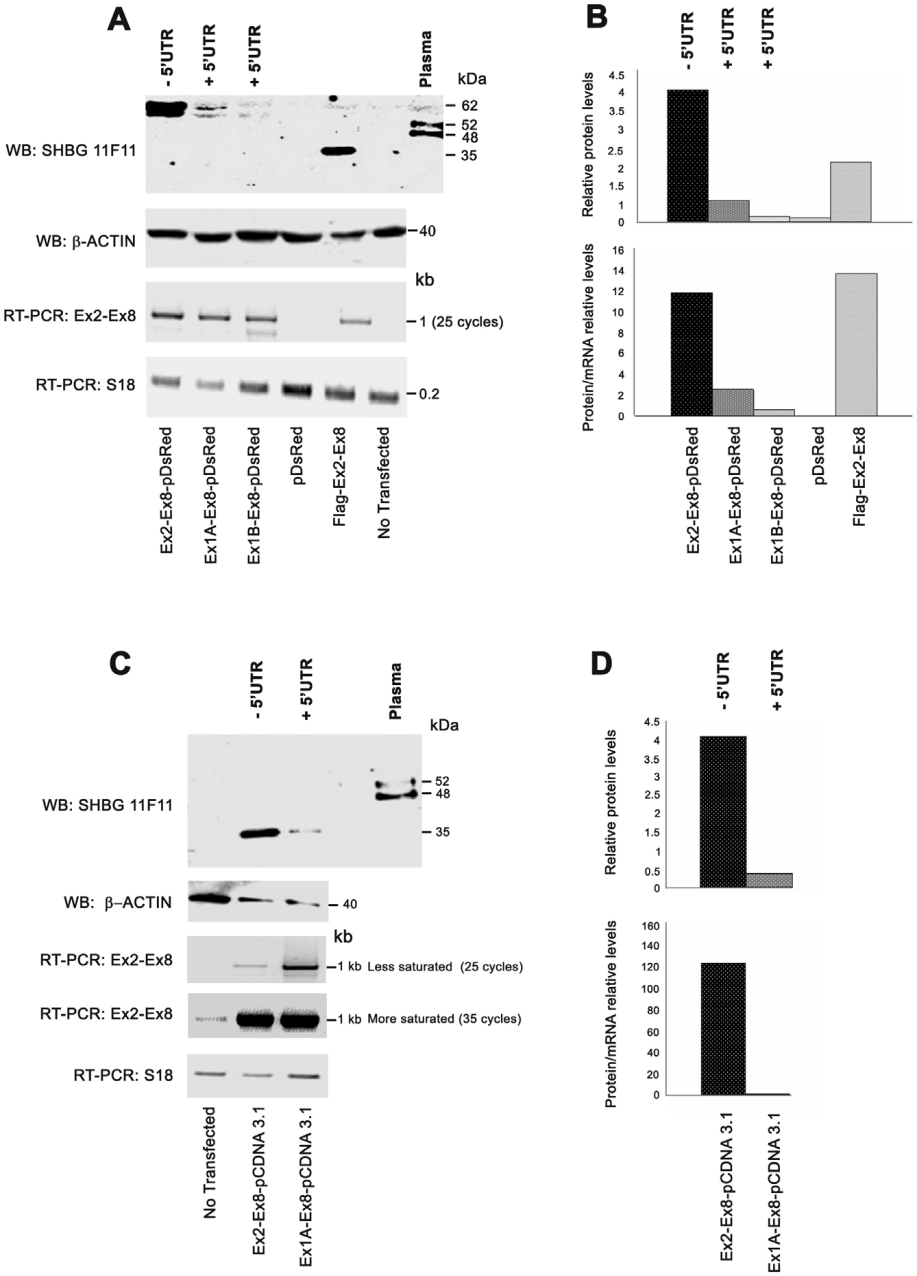
SHBG translation is differentially modulated by alternative exons 1A and 1B. A) Western blot analysis of SHBG translation in LNCaP cells transfected with different SHBG-pDsRed constructs, with and without different 5′UTRs (exon 1A or exon 1B). Human plasma and Flag-SHBG constructs were used as positive controls of SHBG protein detection, while LNCaP cells transfected with pDsRed empty vector and non-transfected cells were used as negative controls. β-actin was used to normalize the quantity of protein loaded on the acrylamide gel. RT-PCR analysis using primers that recognize a region common to all SHBG isoforms (Ex2-Ex8) was performed to normalize the transfection levels with the different SHBG constructs. S18 primers were used to normalize cDNA levels loaded into the PCR reaction. B) Relative protein levels (RPL) and relative protein/mRNA levels (RPML) are shown. RPL was calculated as a ratio of the β-actin level in the sample. RPML was obtained by dividing RPL by the quantified intensity of mRNA SHBG bands. C) Western blot analysis of SHBG translation in LNCaP cells transfected with pcDNA 3-SHBG constructs with and without the 5′UTR (exon 1A). D) RPL and RPML were calculated as above.

We next determined the influence of exons 1A and 1B on SHBG translation. Western blot analysis of LNCaP cells transfected with the SHBG_EX1A/1B-EX8_-pDsRed constructs, which include the exon 1A or 1B 5′UTRs, showed that the amount of SHBG protein was much lower than in cells transfected with the SHBG_EX2-EX8_-pDsRed construct, which did not present any 5′UTRs (Figure 6A, B). Specifically, almost no SHBG was detected in cells transfected with the SHBG_1B-EX8_-pDsRed construct (Figure 6A). The similar amounts of SHBG mRNA in all LNCaP cells transfected with the different SHBG-pDsRed constructs, as determined by SHBG Ex2-Ex8 RT-PCR (Figure 6A), rules out the possibility that variations in SHBG protein levels were due to differences in transcriptional regulation or transfection efficiency. Additionally, LNCaP cells both transfected and not transfected with pDsRed empty vector showed no SHBG immunoreactive band (Figure 6A).

**Figure 7:**
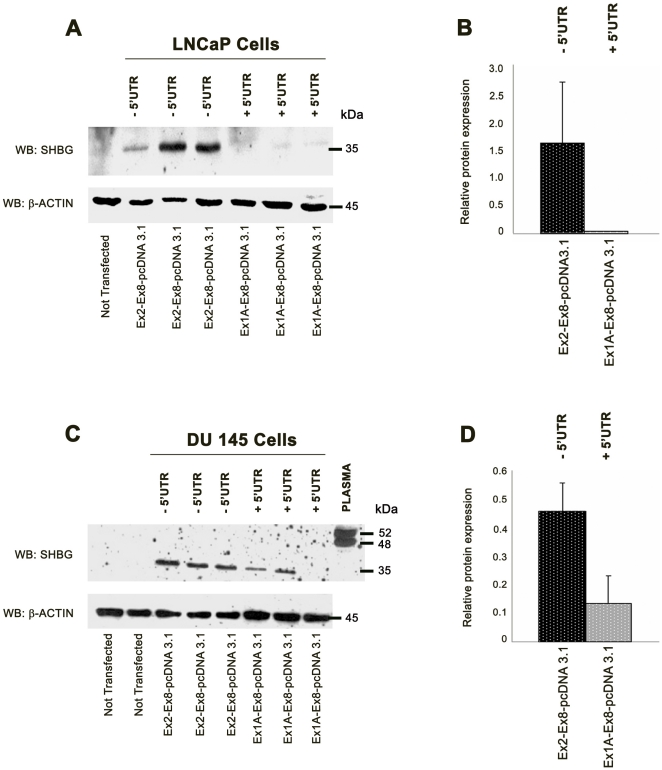
Transfection of pcDNA 3 constructs in LNCaP and DU-145 cell lines. A) Western blot analysis of SHBG translation in LNCaP cells transfected with pcDNA 3-SHBG constructs with and without 5′UTR (exon 1A). Three independent experiments were performed. B) RPL and RPML were calculated. C) Western blot analysis of SHBG translation in DU-145 cells transfected with pcDNA 3-SHBG constructs with and without the 5′UTR (exon 1A). Three independent experiments were performed. D) RPL and RPML were calculated.

Similarly, Western blot analysis of LNCaP transfected cells with 2 different pCDNA3.1 constructs (one including the exon 1A 5′UTR sequence, SHBG_EX1A-EX8_-pCDNA3.1, and the other with no SHBG 5′UTR sequence, SHBG_EX2-EX8_-pCDNA3.1, Figure 5C), showed that cells transfected with the construct containing Ex2-Ex8 produced higher amounts of protein than those transfected with Ex1A-Ex8 (Figure 6C, D). However, analysis of SHBG Ex2-Ex8 mRNA levels by RT-PCR (25 cycles), showed higher amounts of SHBG mRNA in cells transfected with the construct containing Ex1A-Ex8 than in those transfected with the Ex2-Ex8 construct (Figure 6C), indicating than the translation efficiency of Ex1A-Ex8 mRNA was lower than that of Ex2-Ex8 (Figure 6C, D). Additionally, whereas no endogenous SHBG mRNA was detected in non-transfected cells at the exponential phase of the RT-PCR reaction (25 cycles) (Figure 6C), when the number of PCR reaction cycles was extended to 35 (saturating the reaction), the specific band was identified (Figure 6C), indicating that the endogenous SHBG mRNA levels were much lower than those in transfected cells, and therefore, protein levels would be under the limit of detection of the western blot assay (Figure 6A, C). As the molecular size of the SHBG immunoreactive bands was identical in cells transfected with constructs with or without SHBG 5′UTRs (Figure 6A, C), we concluded that the translation start site for exon 1A transcripts was localized in the previously suggested first in-frame ATG of exon 2 (Met 30 of transcripts beginning with the exon 1 sequence). To further support these results, 3 independent transient transfections of the SHBG_EX1A-EX8_-pCDNA3.1 and SHBG_EX2-EX8_-pCDNA3.1 constructs were performed in LNCaP and DU-145 prostate-derived cell lines (Figure 7A, C) and SHBG protein levels were analyzed using polyclonal AF2656 antibody. In both cell lines, SHBG protein levels were again higher in cells transfected with the SHBG-pCDNA construct excluding the exon 1A 5′UTR sequence than in those transfected with constructs including the SHBG 5′UTR (Figure 7A, B, C, D). Thus, the overall results supported that exons 1A and 1B were acting as 5′UTRs that modulate SHBG protein translation.

Application of the CART (classification and regression tree) model established by Davuluri et al. [21] to SHBG 5′UTR sequences of exons 1, 1A and 1B enabled us to classify SHBG first exons into 2 different classes (Table 1): exon 1 belonged to class III (efficiently translated transcripts), whereas exons 1A and 1B belonged to class I (poorly translated transcripts due to the presence of stable secondary structures or uAUGs). None of the SHBG 5′UTRs analyzed corresponded to class II or TOP (5′terminal oligopyrimidines) mRNAs, whose translation is regulated in a growth dependent manner [21].

## Discussion

The SHBG gene is composed of 13 different exons that generate at least 6 different TUs, and a minimum of 19 different transcripts [2], [5]. Each TU is constituted by a common region formed by exons 2 to 8, preceded by one alternative first exon. Only one (exon 1) of the six alternative first exons described (exons 1, 1A, 1B, 1C, 1D, and 1E) presents an ATG in frame with the SHBG coding sequence: TU-1 encodes a leucine-rich signal peptide and is responsible for the production of plasma SHBG by hepatocytes. Translation of TU-1A has been demonstrated in human and mouse sperm containing the 11-kb human SHBG transgene [6], [8], and it has been suggested that TU-1A translation starts at the first in-frame ATG of exon 2, which encodes methionine 30 of transcripts beginning with exon 1 [7], [8]. Translation of TU-1B, -1C, -1D, and -1E has not been previously demonstrated.

The presence of TU-1, -1A, -1B, -1C, -1D, and -1E has been shown in human prostate [2], [5]. We previously demonstrated that TU-1B was the most abundant SHBG TU in the LNCaP, PC3, and PZ-HPV7 cell lines [2], and that transcripts including exon 1 after exon 1A or 1B sequences were also found in prostate cell lines and tissues, indicating that exon 1A/exon 1B and exon 1 can be spliced together and are not always mutually exclusive [2]. Herein, we report the presence of SHBG mRNA corresponding to a common region of all transcription units (Ex2-Ex5) in LNCaP, PC3, and PZ-HPV7 cells, as well as mRNA corresponding to full-length TU-1A and TU-1B in LNCaP cells and human prostate tissue. With regard to their translation, although we were able to identify the SHBG protein in human prostate, testis and plasma, we could not detect its presence in LNCaP, PC3, and PZ-HPV7 cells or in their supernatant, except when SHBG was overexpressed in LNCaP cells with a Flag-tagged SHBG construct. Moreover, when LNCaP cells were transfected with constructs containing the putative non-coding exons 1A or 1B, the amount of detected SHBG decreased considerably with respect to cells transfected with constructs without these potentially noncoding exons. Importantly, the molecular weight of the detected band did not vary between the 2 groups, suggesting that exons 1A and 1B were acting as 5′UTR exons, regulating SHBG translation. These results confirmed that the first in-frame ATG of the exon 2 sequence (which codes for methionine 30 of transcripts beginning with the exon 1 sequence) acts as the first coding codon of TU-1A and TU-1B. In this respect, it has been reported that regulation of translation initiation is a central control point in mammalian cells, and that the rate of initiation limits translation of most mRNAs [22]. Translation regulatory elements in 5′UTRs, such as uAUGs, uORFs and complex mRNA secondary structures [18], are often found in mRNAs encoding regulatory proteins like proto-oncogenes, growth factors and their receptors, and homeodomain proteins [14], [21]. During embryonic development, the 5′UTRs of *Antp, Ubx, RARβ2, c-mos, and c-myc* regulate protein levels in a spatiotemporal manner, and translation initiation of several growth factor mRNAs (*IGFII*, *PDGF2*, *TGFβ*, *FGF-2 and VEGF*) is specifically regulated during differentiation, growth, and stress [21].

The presence of long 5′UTRs containing uAUGs, uORFs, and mRNA secondary structures reduces the efficiency of the scanning process by impeding the ability of ribosomes to interact with the 5′UTR in single-stranded form [18], [21], [22]. Studies in live cells have determined that translation efficiency decreases abruptly when hairpin stabilities in the 5′UTR of genes increase from an dG of −25 kcal/mol to −35 kcal/mol, and reach a basal minimum as hairpins approach predicted thermal stabilities of −50 kcal/mol [19]. In the 5′UTR of TU-1A and TU-1B, we identified 2 uORFs in TU-1A and 1 in TU-1B, as well as thermodynamically stable secondary structures that might intervene in the observed translation downregulation of these transcripts. Furthermore, the greater decrease in translation of TU-1B in comparison with TU-1A might be explained by a higher composition of G/C nucleotides within the 5′UTR sequence of the former, its overall more stable secondary structure, and its longer 5′UTR sequence.

The regulatory role of exons 1A and 1B 5′UTR on SHBG translation has been further confirmed using the CART model for classifying human 5′UTR sequences [6]. According to this classification, the most relevant variables are the presence of TOP (5′ terminal oligopyrimidines), the secondary structure, UTR length, and the existence of uAUGs [21]. Use of the decision tree multivariate analysis of CART for human 5′UTR sequences enabled us to classify transcripts presenting exon 1A-exon 2 and exon 1B-exon 2 5′UTR sequences as poorly translated transcripts (Class I). This class mainly includes mRNAs encoding transcription factors, growth factors, proto-oncogenes, and other regulatory proteins that are poorly translated under normal conditions (eg, in cells in resting state) [21]. The inclusion of exons 1A and 1B 5′UTRs in Class I correlated well with the transient transfection results, in which addition of exon 1A or exon 1B 5′UTR sequences strongly downregulated translation from SHBG Ex2-Ex8 constructs. Additionally, because Class I consists of genes involved in regulation of cell growth and differentiation [21], inclusion of exon 1A 5′UTR in Class I could also explain TU-1A translation in germ cells of mice expressing human SHBG transgene [8]. On the other hand, SHBG TU-1 was classified as efficiently translated transcripts (class III), which also correlated well with easy detection of the secreted SHBG protein in human plasma and in supernatant of HepG2 cell lines. None of the 5′UTRs studied here were classified as class II 5′UTRs which correspond to TOP mRNAs, which are identified by a sequence of 6 to 12 pyrimidines at the 5′end [23].

Selective stress-induced translational control involving uORF has been demonstrated for GCN4, ATF4, ATF5, GADD34, and PKCη [24]. As SHBG exon 1A and 1B transcripts are poorly translated under normal growth conditions, further experiments should be performed to determine whether, under stress conditions, translation of these transcripts is enhanced. Particularly, in prostate cancer, it would be interesting to determine whether the hypoxia environment and the increased oxidative stress associated to tumor growth favor the translation of these alternative transcripts.

Another function of TU-1B might be the regulation of SAT2 gene expression, since it has been described that exon 1B overlaps with the 5′UTR sequence of the SAT2 gene [2], situated on the negative strand of the chromosome 17, and therefore SHBG and SAT2 genes would produce natural sense-antisense pair transcripts that overlap head to head.

Regulation of SHBG translation through its 3′UTR mRNA sequence by miRNA cannot be ruled out. It has been estimated that approximately half of the human genome is controlled by miRNAs, since the human genome contains approximately 1000 miRNAs and each can control up to 10 mRNAs [22]. A large number of in vivo and in vitro studies have shown that miRNAs either inhibit translation, destabilize mRNA, or both [22]. Further studies are required to investigate the contribution of miRNAs to SHBG regulation.

In conclusion, our results in human prostate-derived cell lines indicate that SHBG TU-1A and TU-1B are translated from the first in-frame ATG found in the exon 2 sequence, and that their corresponding 5′UTR exons downregulate SHBG translation.

## Methods

### Cell cultures

All human cell lines were obtained from the American Type Culture Collection (ATCC, Rockville, MD). Prostate cancer cell lines LNCaP, PC3, and DU-145 were maintained in RPMI 1640 medium (PAA Laboratories, Pasching, Austria) containing 10% fetal calf serum (PAA Laboratories) and supplemented with penicillin/streptomycin, sodium pyruvate, and modified Eagle media with nonessential amino acids, as recommended. The hepatocarcinoma cell line HepG2 was maintained in DMEM (PAA laboratories) containing 10% FCS and supplemented as described above. Finally, the prostate cancer cell line PZ-HPV-7 was grown in keratinocyte-SFM medium (Invitrogen, Carlsbad, CA), supplemented with 2.5 µg of EGF and 25 mg of bovine pituitary extract (both from Invitrogen).

### Human prostate tissue

Human prostate tissue was obtained from the non-tumoral part of prostate carcinoma at the T2/T3N0M0 stage of patients submitted to radical prostatectomy. Ethics approval for this study was obtained from the Hospital Universitari Vall d'Hebron Ethics Committee and informed written consent for participation in the study was obtained in all cases, in keeping with the mentioned Committee requirements. The histology of the prostate specimens was evaluated by the urological pathologist.

### Bioinformatics

To predict the secondary structure of SHBG 5′ exons, we used the MFOLD program (version 3.2) (http://mfold.bioinfo.rpi.edu/cg1-bin/rna-form1.cgi) [25] and RNAfold webserver (http://rna.tbi.univie.ac.at/cgi-bin/RNAfold.cgi) from the Vienna RNA websuite [26]. The optimal secondary structures for both sequences were obtained in dot-bracket notation with minimum free energy.

### Generation of SHBG Plasmid Constructs

Three types of constructs were generated: a) a Flag/SHBG construct where the Flag tag is localized at the N-terminus end of the fusion protein (FlagEx2-Ex8); b) two different tag less SHBG pCDNA 3.1 constructs: one including the exon 1A 5′UTR sequence (Ex1A-Ex8-pCDNA3.1), and the other one without any SHBG 5′UTR sequence (Ex2-Ex8-pCDNA3.1); c) three different SHBG/DsRed constructs where the DsRed tag is fused to the C-terminus end of the SHBG protein: two including exon 1A or exon 1B SHBG 5′UTR sequences (Ex1A-Ex8pDsRed or Ex1B-Ex8pDsRed, respectively), and the third one without any SHBG 5′UTR sequence (Ex2-Ex8pDsRed). While the Flag –SHBG construct was used as a positive control of SHBG protein translation as the Flag tag has its own translation start site, both SHBG-pCDNA 3.1 and pDsRed constructs were used to test SHBG protein translation by means of its own translation start codon.

The different SHBG sequences were amplified by PCR from cDNA of a human prostate sample using the primer pairs described in the supplementary table (where the inserted restriction sites are underlined). The PCR products were cloned into the pRC 2.1 vector from the TOPO TA cloning kit (Invitrogen, Carlsbad, CA) and then subcloned into the pCDNA 3.1 vector (kindly provided by Dr. Josep Roma), pDsRed 1N1 (kindly provided by Dr. Maurizio Scaltritti) or pCMV-Flag 6a vectors (Sigma) using the corresponding restriction enzymes (New England Biolabs, Ipswich, MA) (Table S1).

### Transient transfections

One day before transfection, LNCaP and DU 145 cells were seeded in 100-mm culture dishes. The next day, cells were transfected with 4 µg of plasmid DNA (pcDNA3.1-SHBG, SHBG-pDsRed or Flag-SHBG) using Fugene 6 transfection reagent (Roche, Basel, Switzerland), according to a standard protocol. The medium was replaced with fresh medium 16 h post-transfection, and protein and RNA extraction from transfected cells was performed 48 h post-transfection. Transfected cells were examined for SHBG expression by RT-PCR and western blot.

### RNA extraction and RT-PCR

Total RNA was isolated from human cell lines and tissues using the RNeasy Mini/Midi Kit 50 (Qiagen). From each sample, 2 µg of RNA were reverse transcribed using Superscript II H^-^ (Invitrogen), at 42°C for 50 min. One µL of the resulting cDNA was amplified by PCR in non-saturating conditions using the primers described in Table 2. Each PCR was performed in triplicate. The PCR products were resolved by electrophoresis, purified using the QIAquick gel extraction kit (Qiagen), cloned using the TOPO TA cloning system (Invitrogen), and finally sequenced using an ABI Prism 3100 genetic analyzer (Perkin-Elmer Corp., Wellesly, MA).

**Table 2:** List of primers used for RT-PCR.

Primer localization	Primer sequence	*Annealing temperature/cycles*
**Exon 2 upper** **Exon 5 lower**	5′ TGTCATGACCTTTGACCTCACC 3′ 5′ TGAGATCTCGGCCTGTTTGTC 3′	*59°C/35*
**Exon 2 upper** **Exon 8 lower**	5′ TGTCATGACCTTTGACCTCACC 3′ 5′ AGGGGGGTTCTTAGGTGGAGC 3′	*59°C/35*
**Exon 1A upper** **Exon 8 lower**	5′ TTCAAAGGCTCCCCCGCAGTGC 3′5′ AGGGGGGTTCTTAGGTGGAGC 3′	*60°C/39*
**Exon 1B upper** **Exon 8 lower**	5′ TGAAGAGCCTGAGAGAGCG 3′ 5′ AGGGGGGTTCTTAGGTGGAGC 3′	*59°C/39*
***S18 upper*** ***S18 lower***	*5′ GATGGGCGGGGGAAAAT 3′* *5′ CTTGTACTGGCGTGGATTCTGC 3′*	*59–60°C/29*

### Western Blot Analysis

Total protein from human cell lines and tissues was extracted with RIPA buffer containing 150 mM Tris-HCl, 50 mM NaCl, 1% SDS, 1% Nonidet P-40, and 0.5% sodium deoxycholate, complemented with a protease inhibitor cocktail (Sigma). Samples were heat-denatured in loading buffer (Laemmli buffer and DTT) and subjected to discontinuous SDS-PAGE with 4% and 10% polyacrylamide in the stacking and resolving gels, respectively. Proteins were transferred to Trans-Blot nitrocellulose membranes (Biorad, Hercules, CA). Membranes were first blocked for 1 h in TBS containing 0.01% Tween 20 (Sigma) and 5% skim milk, and then incubated overnight at 4°C with primary antibodies against human SHBG (SHBG mouse monoclonal 11F11 antibody, kindly provided by Dr. Geoffrey Hammond; and SHBG goat polyclonal AF2656 antibody, R&D Systems, Minneapolis, MN) in the same buffer. Specific antibody-antigen complexes were identified using horseradish peroxidase-labeled rabbit anti-mouse and goat anti-rabbit IgG secondary antibodies (Dako, Glostrup, Denmark) and then incubated with the chemiluminescent substrate West Dura reagent (Pierce, Etten-Leur, Netherlands). The densitometry was achieved using the Image J software.

To analyze SHBG secretion to the culture media, LNCaP, PC3, PZ-HPV7, and HepG2 cells were seeded in 100-mm culture dishes with the corresponding complete culture medium (see above). After 24 h, the medium was replaced with fresh medium without fetal bovine serum (FBS). Cells were incubated for 48 h with FBS-free medium and 10-mL aliquots of each supernatant were centrifuged in 30-mm×116-mm centrifugal concentrator columns (Sartorius Stedim Biotech, Aubagne, France) at 3000× g for 30 minutes. Each concentrated medium was heat-denatured in loading buffer and analyzed by western blot, as described above.

### Protein deglycosylation

Twenty-five µg of total protein extract were N-deglycosylated using N-glycosidase F enzyme (Roche). Reactions were performed at 37°C O/N using 2 U of enzyme per 10 µg of protein in 0.25 M of Tris-HCl (pH 7–9) in a final volume of 100 µL. Reaction products were analyzed by western blot, as described above.

## Supporting Information

Figure S1 Identification of SHBG 11F11 antibody epitope. LNCaP cells were transfected with several different SHBG contructs; in A and B) cells were transfected with constructs containing the laminin-G-like N-or-C-terminal domains of the SHBG protein tagged with hemagglutinin (HA) or green fluorescent protein (GFP), respectively. Whereas anti-HA and anti-GFP antibodies recognized both constructs, 11F11 antibody only recognized the C-terminal domain. In (C), cells were transfected with Flag-tagged constructs containing: a) the full coding sequence, b) deletion of the last 22 amino acids (351 to 373), and c) deletion of exon 7. In this case, while anti-Flag antibody recognized all the constructs, 11F11 antibody did not recognize the exon 7-deleted construct. Similarly, in D) cells transfected with the GFP-SHBG full-length sequence construct, SHBG was detected by 11F11, whereas the GFP-SHBG construct (exon 7 deleted) was not recognized by the antibody. Therefore, the 11F11 antibody recognizes the C-terminal end of the SHBG protein, and specifically, the region coded by exon 7 and the beginning of exon 8 (up to amino acid 351).
(1.21 MB TIF)

Figure S2 SHBG11F11 antibody epitope is localized at the C-terminal end of the protein. Parallelism between coding exons (black boxes) and the amino acid sequence of SHBG monomer (grey rectangle) is shown. The epitope recognized by 11F11 antibody is localized in the laminin-G-like C-terminal of SHBG protein, specifically in the coded region between all of exon 7 and the beginning of exon 8 (at least to amino acid 351 of mature protein). The two N-glycosylation sites are indicated in black boxes, while the O-glycosylation site is indicated with a black circle.
(0.26 MB TIF)

Table S1 List of primers used to generate SHBG plasmid constructs.
(0.03 MB DOC)
