# Supplementary figures and images for: Human SHBG mRNA Translation Is Modulated by Alternative 5′-Non-Coding Exons 1A and 1B
Source: PLoS One. 2010 Nov 4;5(11):e13844. doi: 10.1371/journal.pone.0013844 (PMC2973947; doi:10.1371/journal.pone.0013844)

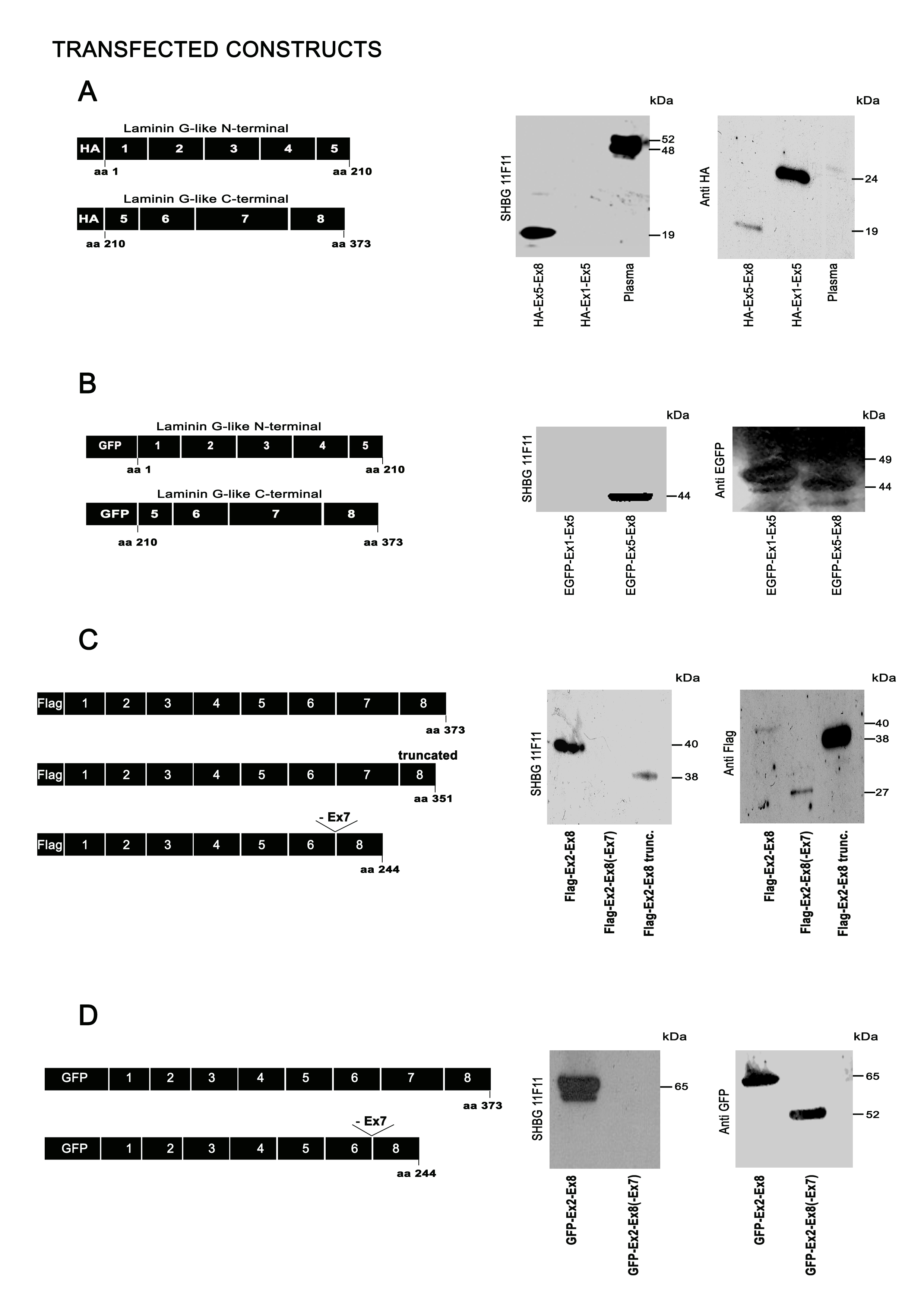

Supplement: Figure S1 — Identification of SHBG 11F11 antibody epitope. LNCaP cells were transfected with several different SHBG contructs; in A and B) cells were transfected with constructs containing the laminin-G-like N-or-C-terminal domains of the SHBG protein tagged with hemagglutinin (HA) or green fluorescent protein (GFP), respectively. Whereas anti-HA and anti-GFP antibodies recognized both constructs, 11F11 antibody only recognized the C-terminal domain. In (C), cells were transfected with Flag-tagged constructs containing: a) the full coding sequence, b) deletion of the last 22 amino acids (351 to 373), and c) deletion of exon 7. In this case, while anti-Flag antibody recognized all the constructs, 11F11 antibody did not recognize the exon 7-deleted construct. Similarly, in D) cells transfected with the GFP-SHBG full-length sequence construct, SHBG was detected by 11F11, whereas the GFP-SHBG construct (exon 7 deleted) was not recognized by the antibody. Therefore, the 11F11 antibody recognizes the C-terminal end of the SHBG protein, and specifically, the region coded by exon 7 and the beginning of exon 8 (up to amino acid 351). (1.21 MB TIF) [file pone.0013844.s001.tif]

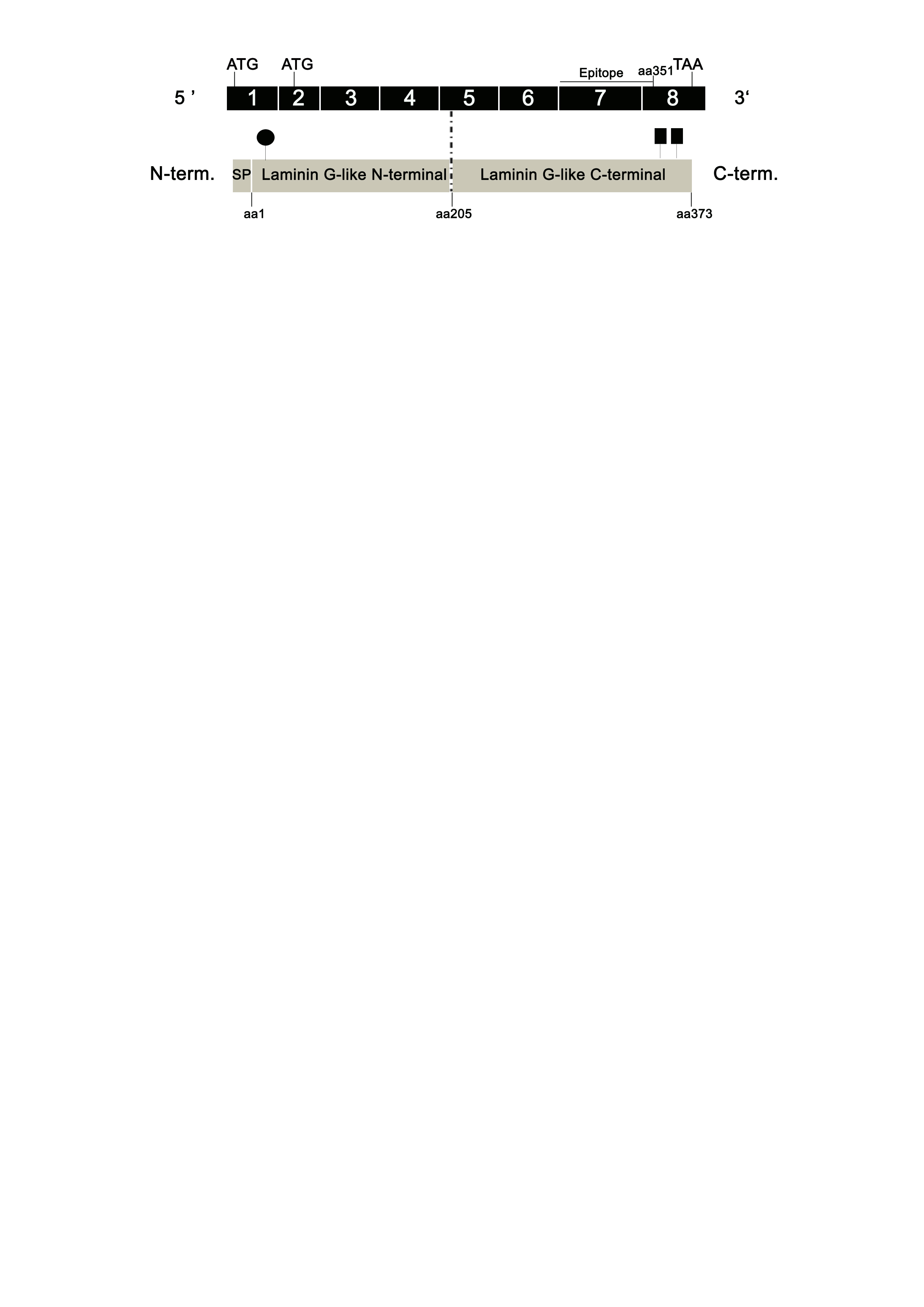

Supplement: Figure S2 — SHBG11F11 antibody epitope is localized at the C-terminal end of the protein. Parallelism between coding exons (black boxes) and the amino acid sequence of SHBG monomer (grey rectangle) is shown. The epitope recognized by 11F11 antibody is localized in the laminin-G-like C-terminal of SHBG protein, specifically in the coded region between all of exon 7 and the beginning of exon 8 (at least to amino acid 351 of mature protein). The two N-glycosylation sites are indicated in black boxes, while the O-glycosylation site is indicated with a black circle. (0.26 MB TIF) [file pone.0013844.s002.tif]
