# Supplementary material for: Human SHBG mRNA Translation Is Modulated by Alternative 5′-Non-Coding Exons 1A and 1B
Source: PLoS One. 2010 Nov 4;5(11):e13844. doi: 10.1371/journal.pone.0013844 (PMC2973947; doi:10.1371/journal.pone.0013844)
Supplement: Table S1 — List of primers used to generate SHBG plasmid constructs. (0.03 MB DOC) [file pone.0013844.s003.doc]

**Primer sequence Restriction enzyme SHBG construct**

5’-CCCAAGCTTGTCATGACCTTTGACCTCACC-3’ *HindIII*-exon 2

Ex2-Ex8SHBG-pcDNA

5’-CCCCTCGAGTCAAGCGTCAGTGCCATTGCCTGG-3’ *XhoI*-exon 8

5’-CCCAAGCTTAAAGGCTCCCCCGCAGTGC-3’ *HindIII*-exon 1A

Ex1A-Ex8SHBG-pcDNA

5’-CCCCTCGAGTCAAGCGTCAGTGCCATTGCCTGG-3 *XhoI*-exon 8

5’-CCCAAGCTTGTCATGACCTTTGACCTCACCAAG-3’ *HindIII*-exon 2

Ex2-Ex8SHBG-pDsRed

5’-CCCGGATCCTGGCTTCTGTTCAGGGCCTG-3’ *BamHI*-exon 8

5’-CCCAAGCTTAAAGGCTCCCCCGCAGTGC-3’ *HindIII*-exon 1A

Ex1A-Ex8SHBG-pDsRed

5’-CCCGGATCCTGGCTTCTGTTCAGGGCCTG-3’ *BamHI*-exon 8

5’-CCCAAGCTTAAGAGCCTGAGAGAGCGG-3’ *HindIII*-exon 1B

Ex1B-Ex8SHBG-pDsRed

5’-CCCGGATCCTGGAAGCGTCAGTGCCATTGCC-3’ *BamHI*-exon 8

5’ CCCAAGCTTGCTGTCATAACCTTTGACCTCACC 3’ *HindIII*-exon 2

Flag-Ex2-Ex8SHBG

5’ CCCCTGGACTCAGGGCCTGGTCCACATCC 3’ *PstI*-exon 8
